# Applications of artificial intelligence in drug discovery

**DOI:** 10.1042/ETLS20250001

**Published:** 2025-11-25

**Authors:** Madhurima Dey, Augustin Amour

**Affiliations:** 1 Discovery Biology Ltd., Cambridge, U.K.; 2 Amour Scientific Consulting Ltd, Cambridge, U.K.

**Keywords:** drug discovery, therapeutics, AI, artificial intelligence

## Abstract

Artificial intelligence (AI) technologies have the potential to revolutionise traditional drug discovery and development. The applications of AI in four prominent areas of drug discovery—drug design, quantum computing, precision medicine and biomarker discovery—are all covered in this issue of Emerging Topics in Life Sciences and summarised in this editorial.

The drug discovery process, which aims to develop biologically active compounds for the treatment of diseases, typically starts with the identification of molecular targets linked to disease biology, followed by the creation and optimisation of active drug candidates that go through several iterations of the Design-Make-Test-Analyse cycle before they become lead candidates entering *in vivo* preclinical development and subsequently clinical trials, ultimately aiming to achieve regulatory approval for administration to patients. However, developing a therapeutic drug traditionally takes 10–17 years, during which average costs range between $100 million and $2 billion, with approximately a 14% success rate of progressing through clinical development and reaching the market, according to the most recent estimates across leading research-based pharmaceutical companies [1]. Notably, the preclinical attrition rates during early discovery stages are typically much greater. Those low success rates are due to the many challenges, developmental hurdles and regulatory milestones in place to ensure scientific robustness and validity before a drug is administered to a patient. Artificial intelligence (AI) technologies, such as machine learning (ML), large language models and generative AI, have the potential to revolutionise traditional drug discovery and development. The convergence of technology and biology into AI/ML models brings the ability to seamlessly integrate multi-modal data and computational power to shorten drug discovery timelines by enhancing the efficiency, accuracy and success rates of scientific research, which has led to significant advancements across various sectors of the industry. The revolutionary potential of the technology is reflected by venture capital funding for AI-driven drug discovery companies, which increased from $30 million in 2013 to a peak of $1.8 billion in 2021 [2].

This issue of *Emerging Topics in Life Sciences* (ETLS) focuses on the applications of AI in four prominent areas of drug discovery: drug design, quantum computing, precision medicine and biomarker discovery. AI-designed therapeutics have not yet reached the market, but the technology is already proving transformative across many stages of drug discovery. Modern drug discovery begins with identifying a disease-relevant target, usually a protein, and developing a molecule that can modulate it. AI/ML algorithms have advanced target discovery by rapidly mining multi-modal, patient-derived omics data (genomics, proteomics, metabolomics, lipidomics) and combining these with causal inference networks to predict drivers of disease biology. Once a target is proposed, its risks, such as the likelihood of adverse effects or uncertainty about its role in disease, are mapped and addressed as early as possible before committing ever-increasing investments throughout the drug discovery process. The process of de-risking a target early, known as target validation, is far from a linear process and therefore does not lend itself well to standardised AI/ML methods. By contrast, the subsequent screening and optimisation of ligands capable of modulating the target, most often small molecules or monoclonal antibodies, follows a more structured Design-Make-Test-Analyse loop where AI/ML is already making major advances and automated pipelines are rapidly becoming incorporated within the process [3].

One of the best-known examples of AI/ML’s impact in preclinical drug discovery is structural modelling, with DeepMind’s AlphaFold transforming protein structure prediction and supporting ligand binding studies to guide drug design. Classical algorithmic approaches such as QSAR (Quantitative Structure–Activity Relationship) have been used for decades to predict ligand affinity, but as Darren Green notes, AI/ML is now driving a new revolution in drug design [4]. Quantum computing is poised to have an impact on drug discovery by enabling quantum simulations with a level of precision that is intractable on classical machines [5]. Examples include AI-enabled quantum annealing for ligand optimisation and quantum molecular dynamics to model enzyme transition states for inhibitor design, which could further transform drug design. Although the field still has a long way to go before reaching its potential, Georg Schusteritsch et al. [6 ] argue that quantum computing, particularly in hybrid workflows, is already extending computational chemistry to more complex problems such as covalent inhibitor design, with tangible near-term impact for drug discovery.

At the far end of the drug discovery process lies the design of clinical trials and the matching of patients to therapies by aligning their omics profiles with an improved therapeutic window, selecting those less likely to experience adverse effects and more likely to benefit. This is the domain of precision or personalised medicine. Andrea Rodriguez-Martinez et al. [7] review how AI/ML is transforming this field by extracting hidden patterns from complex, high-dimensional data to advance disease subtyping and pharmacogenomics, while also highlighting the work that remains to enable widespread clinical adoption. Oncology is leading the way: somatic mutations drive dysregulated signalling across multiple cancers, and rather than pairing tumour type with broad cytotoxic agents, genotypes can now be matched to a growing toolbox of targeted therapies that more precisely address the tumour cell. The same logic can be extended to existing drugs with established safety profiles, enabling repurposing into new indications. As the therapeutic arsenal expands, drug repurposing is likely to become increasingly common.

Precision medicine relies on robust biomarkers, which play a role throughout drug discovery but are most critical in the clinic, where rapid and minimally invasive measures of disease progression and treatment response are essential. A recent example is pTau217, a phosphorylated tau species reflecting neurofibrillary tangles in Alzheimer’s disease that can be measured in blood samples. Roche’s test, which has received FDA Breakthrough Device Designation [8], offers a simple alternative to invasive and costly procedures and is likely to speed up Alzheimer’s clinical trials. As Hira Javaid and collaborators note in this issue of ETLS [9], AI is advancing biomarker discovery through analysis of complex multi-modal datasets, although significant hurdles remain in translating these advances into clinical practice.

AI-enabled technologies have the potential to reveal new frontiers in scientific research and medicine by uncovering novel therapeutics for unmet clinical needs to transform healthcare and patient outcomes. Nonetheless, while the rapid integration of AI across drug discovery has led to scientific advancements, there are a myriad of challenges yet to be overcome before AI can autonomously enhance the process. AI applications are limited by our knowledge and understanding of the complexity of human biology and disease. Furthermore, there are resounding concerns about the rigour, transparency, interpretability and regulation of AI/ML models in drug discovery, as well as ethical dilemmas about privacy and medical data security [10]. As innovation in AI rapidly evolves and the technologies are increasingly incorporated into drug discovery processes, the requirement for collaborative regulatory and legislative frameworks from international institutions, corporations and governing bodies is heightened to maintain scientific rigour and to further enable AI to drive breakthroughs across the health sector. As with other emerging technologies in drug discovery, AI is likely to follow the trajectory described by the Gartner Hype Cycle [11], moving from initial hype through a period of disillusionment before its potential is gradually realised, albeit at an accelerated pace. For now, AI technologies represent an exciting opportunity to augment human-led scientific discovery to address the limitations of traditional drug discovery.

## Open Access

This article has been published open access under our Subscribe to Open programme, made possible through the support of our subscribing institutions, learn more here: https://portlandpress.com/pages/open_access_options_and_prices#conditional

## Abbreviations

AI: artificial intelligence
ETLS: Emerging Topics in Life Sciences
FDA: US Food and Drug Administration
ML: machine learning
pTau217: phosphorylated tau 217
